# T cells conditioned with MDSC show an increased anti-tumor activity after adoptive T cell based immunotherapy

**DOI:** 10.18632/oncotarget.8197

**Published:** 2016-03-19

**Authors:** Patrick L. Raber, Rosa A. Sierra, Paul T. Thevenot, Zhang Shuzhong, Dorota D. Wyczechowska, Takumi Kumai, Esteban Celis, Paulo C. Rodriguez

**Affiliations:** ^1^ Adaptive Biotechnologies, Seattle, WA, USA; ^2^ Georgia Regents University Cancer Center, Augusta, GA, USA; ^3^ Institute of Translational Research, Ochsner Medical Center, New Orleans, LA, USA; ^4^ Stanley S. Scott Cancer Center, Louisiana State University Health Sciences Center, New Orleans, LA, USA

**Keywords:** adoptive T cell transfer immunotherapy (ACT), myeloid-derived suppressor cells (MDSC), mammalian target of rapamycin (mTOR), central memory T cells (T_CM_), stem cell memory T cells (T_SCM_), Immunology and Microbiology Section, Immune response, Immunity

## Abstract

The success of adoptive T cell-based immunotherapy (ACT) in cancer is limited in part by the accumulation of myeloid-derived suppressor cells (MDSC), which block several T cell functions, including T cell proliferation and the expression of various cytotoxic mediators. Paradoxically, the inhibition of CD8^+^ T cell differentiation into cytotoxic populations increased their efficacy after ACT into tumor-bearing hosts. Therefore, we aimed to test the impact of conditioning CD8^+^ T cells with MDSC on their differentiation potential and ACT efficacy. Our results indicate that MDSC impaired the progression of CD8^+^ T cells into effector populations, without altering their activation status, production of IL-2, or signaling through the T cell receptor. In addition, culture of CD8^+^ T cells with MDSC resulted in an increased ACT anti-tumor efficacy, which correlated with a higher frequency of the transferred T cells and elevated IFNγ production. Interestingly, activated CD62L^+^ CD8^+^ Tcells were responsible for the enhanced anti-tumor activity showed by MDSC-exposed T cells. Additional results showed a decreased protein synthesis rate and lower activity of the mammalian/mechanistic target of rapamycin (mTOR) in T cells conditioned with MDSC. Silencing of the negative mTOR regulator tuberous sclerosis complex-2 in T cells co-cultured with MDSC restored mTOR activity, but resulted in T cell apoptosis. These results indicate that conditioning of T cells with MDSC induces stress survival pathways mediated by a blunted mTOR signaling, which regulated T cell differentiation and ACT efficacy. Continuation of this research will enable the development of better strategies to increase ACT responses in cancer.

## INTRODUCTION

Adoptive T cell-based immunotherapy (ACT) to specifically eliminate tumor cells, or to prevent their recurrence, represents an emerging treatment for patients with various malignancies [1, 2]. The success of ACT is significantly limited by the inhibitory inflammatory microenvironment present in tumors [3]. Myeloid-derived suppressor cells (MDSC), a subset of myeloid precursors, are primary components of the tumor milieu and key negative regulators of the function of T cells, natural killer (NK), and dendritic cells [4, 5]. MDSC inhibit T cell immunity through various mechanisms, including the expression of inducible nitric oxide synthase (iNOS) and arginase I [6-8] and the production of nitric oxide (NO), reactive oxygen species (ROS), and peroxynitrite [5]. While MDSC have been closely linked to the inhibition of T cell migration, proliferation, and expression of effector molecules [9], it remains unclear the impact of MDSC on the differentiation of CD8^+^ T cells into effector populations and on T cell efficacy after ACT.

Protective anti-tumor T cell immunity, and therefore the success of ACT, requires differentiation of CD8^+^ T cells into cytolytic and cytokine-producing effector cells [10]. Naïve undifferentiated CD8^+^ CD44^low^ CD62L^+^ T cells initially progress into CD8^+^ CD44^high^ CD62L^+^ central memory T cells (T_CM_), and then into CD8^+^ CD44^high^ CD62L^−^ effector memory T cells (T_EM_) and effector T cells (T_EFF_) [11]. Paradoxically, acquisition of full effector function before ACT impairs the anti-tumor effect of CD8^+^ T cells [12]. In fact, transfer of activated undifferentiated CD44^low^ CD62L^+^ CD8^+^ T cells (referred as stem cell memory T cells (T_SCM_)) into tumor-bearing mice resulted in higher anti-tumor responses, compared to those found in mice receiving T_EM_ cells [13]. Thus, inhibition of CD8^+^ T cell differentiation increased their efficacy after ACT.

Multiple pathways regulate the metabolically demanding process of differentiation of activated CD8^+^ T cells into terminal T_EM_ cells, including the activation of the mammalian/mechanistic target of rapamycin complex I (mTOR) [14, 15]. Among different cellular effects, mTOR regulates global protein synthesis through the phosphorylation of the translation initiation factor P70-S6K and the inactivation of the translation inhibitor eIF4E-binding protein (4E-BP1) [16]. Under favorable cellular conditions, mTOR signaling induces cap-dependent translation and ribosomal assembly. However, after exposure of cells to stress conditions, mTOR activity drops, allowing cells to adapt to the harsh environment [17]. Recent studies showed that the inhibition of mTOR in T cells enhanced their survival and anti-tumor activity after ACT [18]. However, the effect of MDSC on T cell mTOR activity and thereby on ACT remains unknown.

Here, we sought to determine the effect of MDSC in the differentiation and anti-tumor activity of CD8^+^ T cells used for ACT. Our findings indicate that transient conditioning of T cells with MDSC inhibits their differentiation into effector T cells and improves their anti-tumor efficacy after ACT. These effects correlated with a lower mTOR signaling, which regulated the survival of T cells after exposure to MDSC. Continuation of this research could provide beneficial implications for ACT approaches in cancer patients.

## RESULTS

### MDSC impair the differentiation of *in vitro* activated CD8^+^ T cells independently of TCR signaling

**Figure 1:**
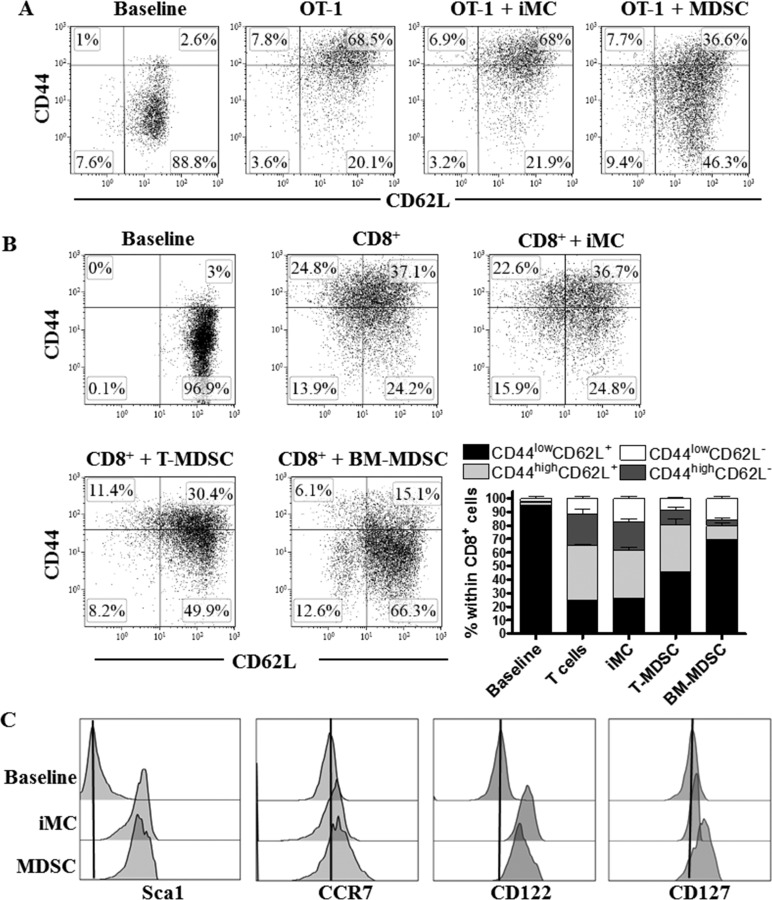
MDSC impairs activated CD8^+^ T cell differentiation **A.** OT-1 cells were activated with SIINFEKL (2 μg/ml) and cultured alone or in the presence of iMC or tumor-MDSC (1:1/2) for 48 hours, after which CD8^+^ T cells were tested by flow cytometry for the expression of CD62L and CD44. Baseline represented the non-stimulated CD8^+^ T cells. Dot plots are from 3 repeats. **B.** CD8^+^ T cells were stimulated with anti-CD3-CD28 and cultured alone or in the presence of iMC, BM-MDSC or tumor-MDSC. The expression of CD62L and CD44 within gated CD8^+^ T cells was monitored 72 hours later by flow cytometry. Bars, represent mean +/− SEM from 3 experiments. **C.** Expression of Sca1, CCR7, CD122, and CD127 was tested by flow cytometry in gated CD8^+^ CD44^low^ CD62L^+^ cells from non-activated T cells (baseline) or activated T cells (anti-CD3-CD28) co-cultured with iMC or MDSC for 72 hours. Histograms are a representative finding from 3 separate repeats.

We first sought to determine whether MDSC altered the *in vitro* progression of activated CD8^+^ T cells into effector populations. To test this, we monitored the expression of the differentiation markers, CD44 and CD62L, in SIINFEKL-activated CD8^+^ T cells from OT-1 mice co-cultured with tumor-MDSC or non-suppressive immature myeloid cells (iMC). The expression of CD44 increases as CD8^+^ T cells differentiate into T_EFF_ cells, whereas CD62L levels are progressively lost [19]. An elevated percentage of undifferentiated CD44^low^ CD62L^+^ CD8^+^ T cells was found in SIINFEKL-primed OT-1 cells treated with MDSC, compared to those exposed to iMC, which progressed mainly into CD44^high^ CD62L^+^ T_CM_ cells (Figure 1A). Also, a similar CD44^low^ CD62L^+^ arrest was observed in CD8^+^ T cells activated with anti-CD3/CD28 and co-cultured with tumor-MDSC or bone marrow-derived MDSC (BM-MDSC) (Figure 1B), confirming the inhibitory effect of MDSC on T_EFF_ differentiation. Because naïve and undifferentiated primed CD8^+^ T cells share the phenotype CD44^low^ CD62L^+^ [11], and MDSC significantly blunted proliferation of activated CD8^+^ T cells (Suppl. Figure 1), we studied whether MDSC blocked the activation of CD8^+^ T cells. A similar increase in the activation-T_SCM_ markers Sca-1, CCR7, CD122, and CD127 was noted in CD44^low^ CD62L^+^ T cells co-cultured with MDSC or iMC, but not in resting T cells (Figure 1C), indicating that the CD44^low^ CD62L^+^ phenotype induced by MDSC was distinct from that of naïve T cells.

**Figure 2:**
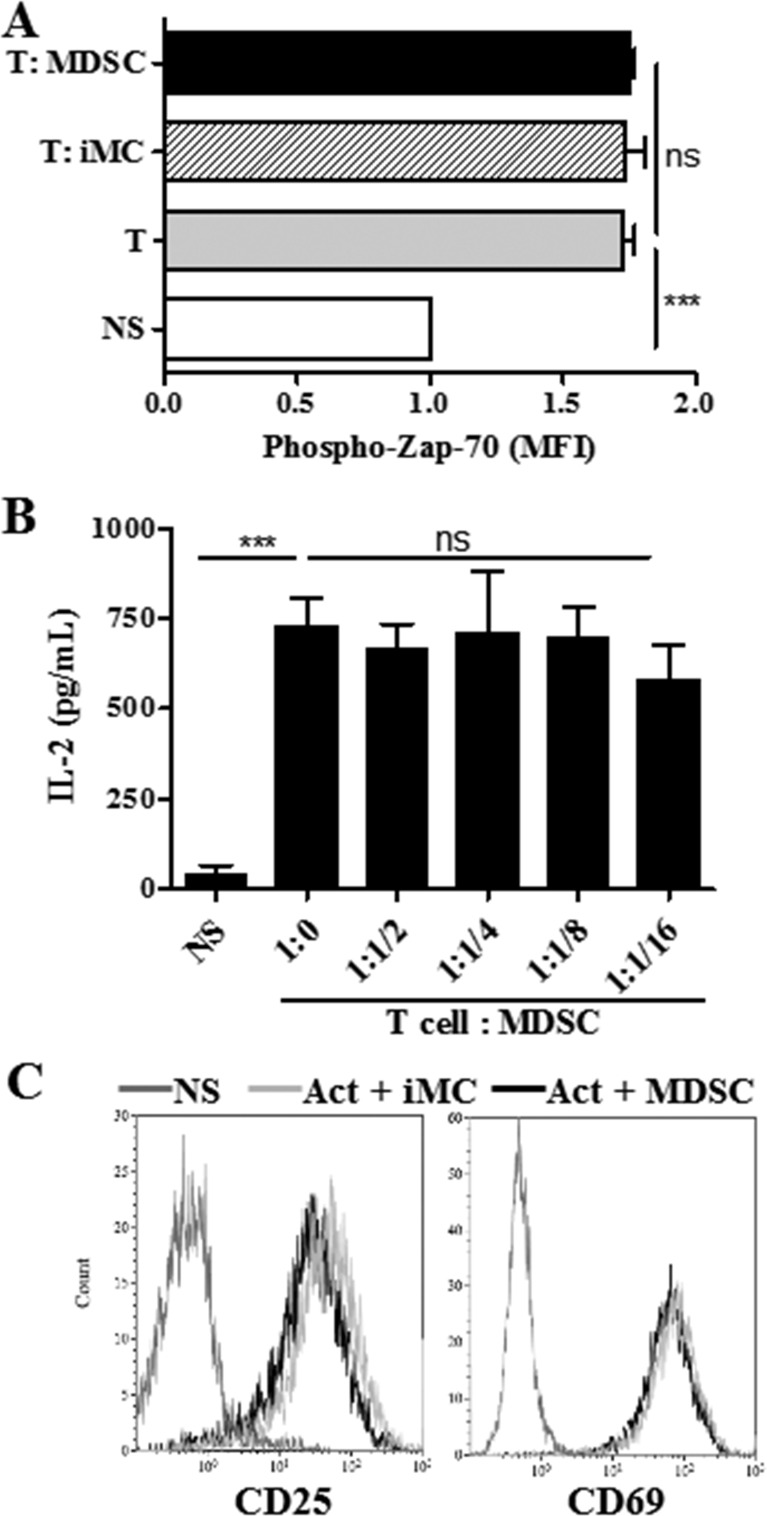
MDSC does not impair T cell receptor associated signaling **A.** Intracellular pY319-Zap-70 levels were assessed by flow cytometry in non-stimulated (NS) or activated T cells cultured alone or in the presence of iMC or MDSC (ratio 1:1/2). Bars are mean +/− SEM of mean fluorescence intensity (MFI) from 2 repeats. **B.** IL-2 levels measured by ELISA in supernatants from activated T cells cultured alone or in the presence of increasing MDSC numbers. Non-activated T cells (NS). Data are expressed as mean +/− SEM from 3 experiments. Expression of early activation markers CD25 and CD69 in activated T cells co-cultured for 24 hours with iMC or MDSC. Values are from 3 repeats. ***p < 0.001, ns *p*> non-significant.

To further assess the effect of tumor-MDSC on early stages of T cell activation, we measured the expression of phospho-Zap-70 (pY319), a major kinase related with early stages of T cell receptor (TCR) signaling [20]. Co-culture of T cells with MDSC did not impair the upregulation of phospho-Zap-70 induced upon anti-CD3/CD28 activation (Figure 2A). In addition, equivalent levels of IL-2 and a similar induction of early activation markers CD25 and CD69 were found in control activated T cells and those co-cultured with tumor-MDSC (Figure 2B-2C), demonstrating that MDSC impaired the progression of CD8^+^ T cells into effector populations, without altering TCR-related signaling or early activation processes.

### Arrest on CD8^+^ T cell differentiation by MDSC depends on permanent cell to cell contact

**Figure 3:**
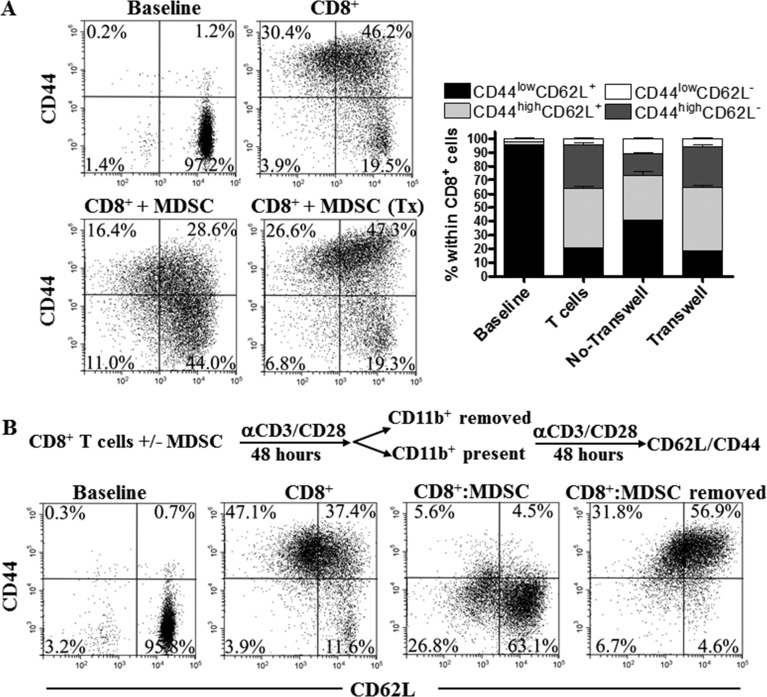
MDSC impair CD8^+^ T cell differentiation through permanent cell to cell contact **A.** 1×10^6^ CD8^+^ T cells were activated with anti-CD3/CD28 and cultured with MDSC (1:1/2) using or not transwells. The levels of CD44 and CD62L were then monitored 72 hours later. Dot plots are a representation of one of 3 similar experiments. Bars represent mean +/− SEM from 3 experiments. **B.** Activated CD8^+^ T cells were cultured alone or with tumor-MDSC for 48 hours. Then, MDSC were depleted using anti-CD11b beads and the CD8^+^ T cells cultured alone or with MDSC for additional 48 hours, after which the expression of CD44 and CD62L was established by flow cytometry. Figures are a representative dot plot from 3 independent and similar repeats.

We studied the need for cell to cell contact during the arrest on T cell differentiation by MDSC. An increased percentage of CD44^low^ CD62L^+^ cells was found in activated CD8^+^ T cells directly co-cultured with MDSC, but not in those separated by transwells (Figure 3A), indicating that the arrest in CD8^+^ T cell differentiation induced by MDSC relies on close proximity between MDSC and T cells. This agrees with previous reports showing the close contact requirement for the T cell suppression mediated by MDSC-related reactive nitrogen species [21-23]. Then, we investigated whether the alterations in CD8^+^ T cell differentiation triggered by tumor-MDSC were permanent. Deletion of MDSC after 48 hours of T:MDSC co-culture restored the differentiation of CD8^+^ T cells into a CD44^high^ CD62L^+^ phenotype (Figure 3B), suggesting that the differentiation arrest induced by MDSC was a reversible process.

### T cells conditioned with MDSC display an increased anti-tumor efficacy after ACT and become less susceptible to MDSC upon re-exposure

**Figure 4:**
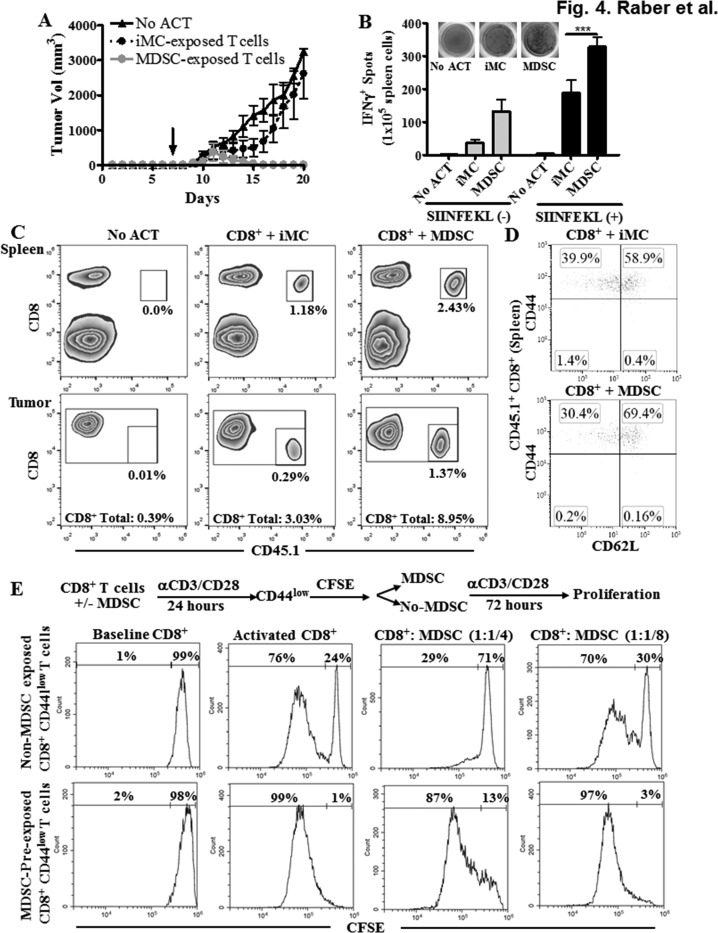
T cells conditioned with MDSC show an increased anti-tumor efficacy after ACT **A.** CD45.2^+^ C57BL/6 mice were injected s.c. with EG-7 cells for 7 days to develop palpable tumors. Next, mice received via tail vein 1×10^6^ CD8^+^ CD45.1^+^ OT-l T cells activated with SIINFEKL (2 μg/mL) and cultured alone or in the presence of iMC or MDSC for 48 hours prior to injection. Tumor volume was then monitored until endpoint. Mice that did not receive OT-1 cells (no ACT) served as controls. *n* = 5 mice per condition. ***p < 0.001 **B.** Fourteen days after the ACT, mice were sacrificed and the spleens from each group analyzed by Elispot for IFNγ production upon overnight challenge with SIINFEKL. **C.** Spleens and tumors from mice **B.** were analyzed for the accumulation of CD45.1^+^ CD8^+^ cells. *n* = 5 per group. **D.** Expression of CD62L and CD44 was monitored in the spleens of mice from **B.** after gating within the transferred OT-1 cells (CD45.1^+^ CD8^+^). Figures are a representative dot plot from 5 tumor-bearing mice per group. **E.** Activated CD8^+^ T cells were cultured with MDSC (1:1) for 24 hours, after which the CD44^low^ CD8^+^ T cells were sorted, labeled with CFSE (baseline), and cultured alone or in the presence of MDSC (1:1/4; 1:1/8) for additional 72 hours. CFSE dilution was then monitored by flow cytometry.

Transfer of CD44^low^ CD62L^+^ CD8^+^ T cells into tumor-bearing mice resulted in higher anti-tumor responses [11]. Therefore, we studied whether antigen-specific CD8^+^ T cells expanded in the presence of MDSC exhibited a higher anti-tumor activity after ACT. Splenocytes from CD45.1^+^ OT-1 mice were activated with SIINFEKL and co-cultured with MDSC or iMC for 48 hours, after which CD8^+^ T cells were sorted and transferred into CD45.2^+^ mice bearing s.c. EG7 tumors for 7 days. A higher anti-tumor effect was found in EG7-bearing mice transferred with MDSC-conditioned CD8^+^ T cells compared to those receiving iMC-treated CD8^+^ T cells (Figure 4A). The increased anti-tumor efficacy induced by MDSC-educated OT-1 cells correlated with a higher IFNγ production and an elevated frequency of the transferred CD8^+^ T cells in spleen and tumor (Figure 4B-4C). Thus, expansion of CD8^+^ T cells in the presence of MDSC improved ACT efficacy. Next, we studied whether T cells pre-exposed to MDSC maintained their phenotype after transfer into EG7-bearing mice. A lower frequency of terminal T_EFF_ CD44^high^ CD62L^−^ cells, and a higher percentage of T_CM_ CD44^high^ CD62L^+^ T cells were found in the transferred CD45.1^+^ CD8^+^ OT-1 cells conditioned with MDSC, compared to those exposed to iMC (Figure 4D), suggesting that initial culture of CD8^+^ T cells with MDSC delays their differentiation into T_EFF_ *in vivo*.

Next, we investigated whether the CD44^low^ CD8^+^ T cells previously conditioned with MDSC became less susceptible to MDSC upon re-exposure. Thus, anti-CD3/CD28 primed CD8^+^ T cells were co-cultured with or without MDSC for 24 hours, after which CD44^low^ CD8^+^ T cells were sorted, labeled with CFSE, and cultured alone or in the presence of MDSC for additional 72 hours. Initial exposure of activated CD8^+^ T cells to MDSC significantly increased the ability of CD44^low^ CD8^+^ T cells to proliferate both alone and in the presence of MDSC, compared to CD44^low^ CD8^+^ T cells initially cultured in the absence of MDSC (Figure 4E, Suppl. Figure 2). Thus, our results indicate that MDSC-educated CD44^low^ CD8^+^ T cells displayed a higher proliferative potential and became less susceptible to MDSC upon re-exposure.

### Role of CD62L^+^ T cells in the anti-tumor effects induced by MDSC-educated T cells

**Figure 5:**
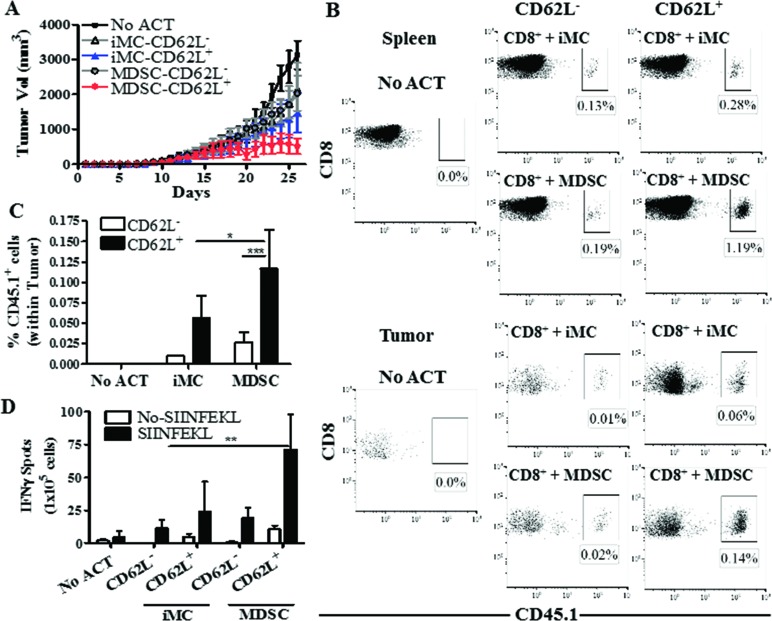
MDSC-conditioned CD62L^+^ T cells show higher anti-tumor efficacy **A.** CD45.1^+^ CD8^+^ OT-l T cells activated with SIINFEKL (2 μg/mL) and cultured for 48 hours in the presence of iMC or MDSC were sorted by flow cytometry into CD62L^+^ and CD62L^−^ cells. The sorted populations were then transferred (4×10^5^/mouse) into CD45.2^+^ C57BL/6 mice bearing established EG7 tumors, after which tumor burden was measured until endpoint. Mice that did not receive OT-1 cells served as controls (No ACT). *n* = 5 mice per group. **B.** Twelve days following the ACT, mice were sacrificed and spleens and tumors analyzed for the accumulation of the transferred CD45.1^+^ CD8^+^ cells. Figure is a representation of results from 5 different mice. **C.** Quantitation of the accumulation of CD45.1^+^ CD8^+^ cells after ACT in the tumors. Data are the mean +/− SEM from 3 experiments. ***p < 0.001. **D.** Spleens from each group were analyzed by Elispot for IFNγ production after overnight challenge with SIINFEKL. *n* = 5 mice. **p < 0.01

We aimed to determine whether the CD44^low^ CD62L^+^ T cells were responsible for the enhanced anti-tumor effects induced by MDSC-conditioned T cells. However, we were limited by the low proliferation of T cells co-cultured with MDSC, which did not allow to sort enough numbers of CD44^low^ CD62L^+^ T cells for the ACT. Alternatively, we sorted CD62L^−^ and CD62L^+^ T cells and tested their role in the anti-tumor activity induced by MDSC-educated T cells. SIINFEKL-activated CD45.1^+^ OT-1 cells were cultured for 48 hours with MDSC or iMC, after which CD8^+^ T cells were sorted by flow cytometry into CD62L^+^ and CD62L^−^ cells and transferred i.v. into CD45.2^+^ mice bearing established s.c. EG7 tumors. A significant delay in EG7 tumor growth was found in mice transferred with MDSC-treated CD62L^+^ T cells, compared to those receiving MDSC-exposed CD62L^−^ counterparts or iMC-conditioned T cells (Figure 5A). Accordingly, transferred-CD62L^+^ CD8^+^ T cells expanded in the presence of MDSC showed a higher accumulation in tumor and spleen (Figure 5B-5C) and had an elevated IFNγ production (Figure 5D), compared to the counterpart groups of transferred CD8^+^ T cells. Thus, our results suggest that CD62L^+^ T cells were responsible for the elevated anti-tumor effects induced by MDSC-conditioned T cells.

### MDSC impair global translation and mTOR signaling in T cells

**Figure 6:**
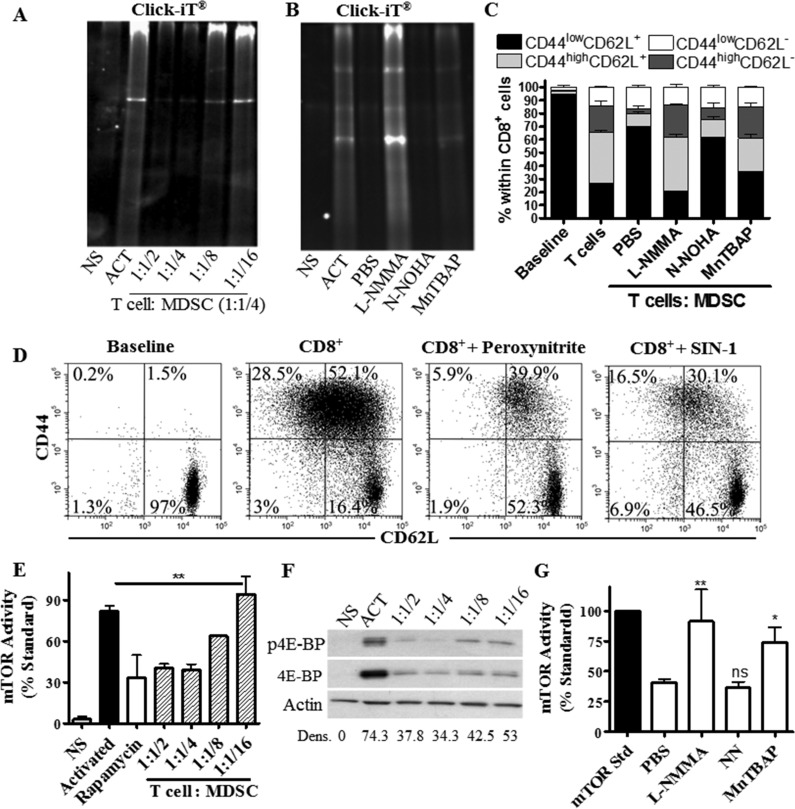
T cells limit *de novo* translation and mTOR activity in response to MDSC **A.**-**B.** Activated T cells were cultured alone (Act) or with increasing numbers of MDSC **A.**, or at a fixed ratio of 4:1 (T cell : MDSC) in the presence of L-NMMA (500μM), N-NOHA (200μM), or MnTBAP (100μM) **B.** *De novo* protein synthesis was then monitored in T cells using Click-iT as described in the methods. NS indicates non-activated T cells. Figures are a representative result of 3 repeats. **C.** Activated CD8^+^ T cells were cultured for 72 hours in the presence of MDSC, as well as L-NMMA, N-NOHA, or MnTBAP. Then, CD62L and CD44 expression was monitored in gated CD8^+^ T cells by flow cytometry. Data are mean +/− SEM from 3 experiments. ***p < 0.001. **D.** Expression of CD62L and CD44 in CD8^+^ T cells activated with anti-CD3/CD28 and cultured with peroxynitrite (100 μM) or SIN-1 (0.1 mM) for 72 hours. Results are a representative experiment of 3 repeats. **E.**-**G.** Cellular lysates from activated T cells cultured alone or in the presence of MDSC were analyzed for mTOR activity **E.** or the expression of phosphorylated and total 4E-BP1 **F.** Gray bar represents non-activated T cells (NS); black bar represents activated T cells; white bar is activated T cells cultured with rapamycin (100 ng/ml), striped bars are activated T cells co-cultured with MDSC. **G.** Lysates from (B) were tested for mTOR activity. Values are expressed as mean +/− SEM from 3 experiments.

Inhibition of protein synthesis pathways has been shown to block the progression of CD8^+^ T cells into effector populations [18]. We asked whether MDSC could regulate *de novo* translation in stimulated CD8^+^ T cells. Using the non-radioactive method Click-iT^®^ and [^35^S] Methionine, we found that MDSC severely restricted *de novo* protein synthesis in primed T cells (Figure 6A, Suppl. Figure 3A). This effect correlated with markers of a blunted T cell-translation [24], including an arrest in the G_0_-G_1_ phase of the cell cycle and an inhibited expression of cyclin D3 and cdk4 (Suppl. Figure 3B-3C). Interestingly, arginase inhibition by Nor-NOHA [7] did not affect the impaired protein translation and differentiation arrest induced by MDSC on T cells (Figure 6B-6C). In contrast, inhibition of iNOS through L-NMMA completely restored *de novo* protein synthesis and T cell differentiation in activated CD8^+^ T cells co-cultured with MDSC, while the scavenging of peroxynitrite using MnTBAP [25] induced a partial rescue effect (Figure 6B-6C, Suppl. Figure 3A). Furthermore, treatment of CD8^+^ T cells with non-toxic doses of peroxynitrite or nitric oxide donor SIN-1 resulted in an arrested CD8^+^ T cell differentiation (Figure 6D). These results indicate a potential role of reactive nitrogen species on the T cell differentiation alterations induced by MDSC.

mTOR promotes *de novo* protein synthesis through the phosphorylation of the translation initiation inducer P70-S6K and inactivation of the translation inhibitor 4E-BP1 [16]. Because MDSC impaired *de novo* translation in T cells, we determined the potential role of a decreased mTOR signaling in this process. Using an activity method that measures the ability of mTOR to phosphorylate P70-S6K, we found that MDSC inhibited mTOR signaling in stimulated T cells to those levels found in T cells treated with mTOR inhibitor rapamycin (Figure 6E). In addition, a lower expression of mTOR target 4E-BP1 was found in activated T cells cultured with MDSC, compared to control stimulated T cells (Figure 6F). Furthermore, we found that inhibition of iNOS or scavenging of peroxynitrite restored mTOR activity in T cells treated with MDSC (Figure 6G), indicating a key role of reactive nitrogen species in the inhibition of T cell-mTOR by MDSC.

### Overcoming mTOR inhibition in T cells co-cultured with MDSC results in T cell apoptosis

**Figure 7:**
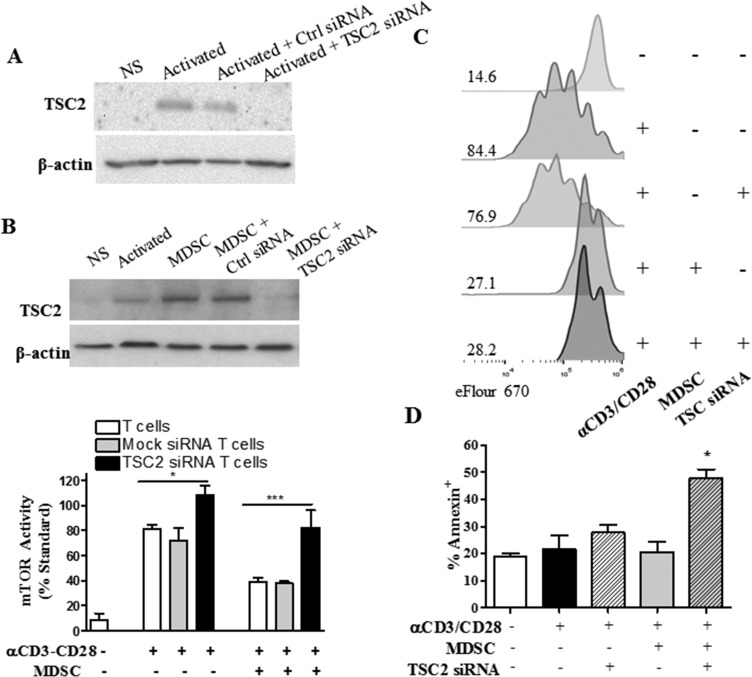
TSC2-silencing restores mTOR activity in T cells co-cultured with MDSC, but results in T cell apoptosis **A.** A representative experiment showing the expression of TSC2 in lysates from activated T cells transfected with control or TSC2 siRNA for 72 hours. **B.** TSC2 expression (top) and mTOR activity (bottom) in mock and TSC2 siRNA-transfected T cells cultured with MDSC for 72 hours. **C.** eFluor670 dilution was analyzed by flow cytometry in activated T cells co-cultured with MDSC and/or transfected with TSC2 specific siRNA. Histogram figures are a representative result from 3 experiments. **D.** T cells from (B) were monitored for the expression of Annexin V by flow cytometry. All results are from a minimum of three independent repeats.

To determine the effect of overcoming the mTOR inhibition in T cells co-cultured with MDSC, we aimed to silence the expression of tuberous sclerosis complex 2 (TSC2) in T cells (Figure 7A), a key negative regulator of mTOR signaling [16]. Silencing of TSC2 enhanced mTOR activity in stimulated T cells cultured alone and completely restored mTOR activity in T cells co-cultured with MDSC (Figure 7B). However, we could not determine the effect of restoring mTOR signaling in the differentiation of MDSC-conditioned T cells, as TSC2 silencing in T cells co-cultured with MDSC did not restore proliferation and resulted in a significant induction of the apoptosis marker Annexin V (Figure 7C-7D). Interestingly, the increased apoptosis was not observed in T cells carrying a mock siRNA and co-cultured with MDSC, suggesting a key role of the inhibition of mTOR signaling in the survival of T cells exposed to MDSC. Altogether, our results indicate that conditioning of T cells with MDSC induces survival pathways characterized by a blunted mTOR signaling, which prevents T cell differentiation and increases the anti-tumor efficacy of ACT.

## DISCUSSION

Protective anti-tumor T cell responses require the proliferation and differentiation of CD8^+^ T cells into cytotoxic populations [26]. Paradoxically, transfer of undifferentiated CD8^+^ T cells into tumor-bearing mice resulted in higher anti-tumor responses and an extended survival of the transferred cells [12]. Thus, we tested whether the expansion of T cells in the presence of MDSC altered their differentiation into cytolytic T cells and impacted their anti-tumor effects after ACT. Our results show that conditioning of T cells with MDSC inhibited their progression into effector populations and enhanced their ability to induce anti-tumor effects upon ACT. This surprising finding could represent a novel approach for the improvement of ACT strategies in cancer.

The traditional focus of the relationship between MDSC and T cells is that of suppression. As such, the vast body of work on the subject primarily describes the negative impact of MDSC on anti-tumor immunity and on the efficacy of cancer immunotherapies [27]. Here, we suggest the potential for MDSC-associated suppressive mechanisms to serve as a means to improve ACT through the inhibition of effector T cell differentiation. Although this approach elevated the anti-tumor activity of T cells, it remains unclear whether this transient MDSC exposure rendered T cells resistant to the suppressive tumor microenvironment or to the MDSC present in tumors. As such, the initial results presented in this manuscript show that conditioning of CD8^+^ T cells with MDSC decreased the susceptibility of CD44^low^ CD8^+^ T cells to MDSC upon re-exposure. However, further studies specifically testing the resistance of this population to MDSC in tumor-bearing mice need to be performed. Furthermore, our results together with previous publications [26, 28] indicate that the induction of transient stress pathways in T cells could eventually increase their ability to effectively control tumor growth. Although this effect would be hardly achieved under physiological conditions, it could represent a therapeutic opportunity to increase the efficacy of ACT approaches in cancer. In fact, transient exposure to peroxynitrite or nitric oxide donors could enable the use of this effect in therapeutic models.

Activation of the Wnt-β-catenin pathway in T cells induced a T_SCM_ phenotype characterized by expression of CD44^low^ CD62L^high^ Sca-1^high^ CD122^high^ and an increased capacity to survive and self-renew in tumor-bearing mice [19]. This promotion of T_SCM_ was also noted after treatment of acute lymphocytic choriomeningitis-infected mice with rapamycin, which significantly improved the quantity and quality of virus-specific memory CD8^+^ T cells [14]. Also, inhibition of mTOR using T cell-specific aptamers delivering mTORC1 specific siRNA or by Temsirolimus resulted in an enhanced anti-tumor effector response and vaccine-derived immunity [18, 29]. These results are in accordance with our findings indicating a potential role of the inhibition of T cell-mTOR by MDSC in the decreased differentiation of CD8^+^ T cells into effector populations. However, the specific mechanisms by which MDSC regulate mTOR activity remain unknown. Our data suggest a potential effect of the release of reactive nitrogen species by MDSC in the regulation of mTOR signaling in T cells. In fact, previous reports showed that nitrosylation of mTOR components regulates overall mTOR activity [30]. Silencing of TSC2 restored mTOR activity in T cells cultured with MDSC, but it also led to T cell apoptosis, suggesting the key role of this inhibition in the survival of T cells to MDSC. However, the mechanisms by which mTOR inhibition prevents cell death in T cells co-cultured with MDSC remain completely unknown. Further elucidation of the pathways by which MDSC regulate mTOR in T cells may prove essential for improving T cell responses in future therapies and for the development of treatments to overcome the suppression of T cells in patients with cancer.

The notion that T cells are primed and ready to fight tumors, but are limited by the tumor microenvironment has been demonstrated in patients with melanoma in which lymphocytes from excised tumor masses will expand when supplemented with IL-2 [31]. Our results show that T cells will limit mTOR signaling as a means of coping with the suppressive effect induced by MDSC. These T cells are essentially primed, but waiting for the favorable conditions before entering the laborious metabolic process of cellular division and progression into effector cells. The idea that MDSC can promote stemness in T cells by reducing mTOR signaling is somewhat unorthodox but not unjustified, as the mTOR pathway evolved as a means of integrating multiple cellular signals. This allows mTOR to drive T cell function but also to stand as sentinel during periods of stress, including changes in nutrient availability, high levels of oxidative stress, and hypoxia. In fact, while treatment with mTOR inhibitors induced T_SCM_ phenotypes and favored anti-tumor responses in some models, it also promoted the development of MDSC, regulatory T cells (Tregs), and tolerogenic dendritic cells [32]. Moreover, rapamycin treatment prevented the protective CD8^+^ T cell responses induced by a human papilloma virus E7 peptide vaccine in tumor-bearing mice [33]. This effect could be induced by Tregs induced by mTOR inhibition [34].

In summary, our results show that T cells conditioned with MDSC show an increased anti-tumor activity after ACT, which correlated with a low progression of cells into effector populations and an arrest in mTOR signaling. Continuation of this research could bring beneficial implications for ACT approaches in cancer as it may demonstrate a new strategy for priming T cells prior to therapeutic transfer.

## MATERIALS AND METHODS

### Animals and cell lines

Lewis lung carcinoma (3LL) and EL-4 thymoma expressing ovalbumin (EG7) were maintained in RPMI-1640 (Lonza-Biowhittaker, Walkerville, MD) and supplemented with 10% fetal calf serum (Hyclone, Logan, UT), 25 mM Hepes (Invitrogen, Life Technologies, Grand Island, NY), 4 mM L-glutamine (Invitrogen, Life Technologies), and 100 U/mL of penicillin, streptomycin (Invitrogen, Life Technologies). Cell lines were obtained from the American Type Culture Collection (ATCC, Manassas, VA) and periodically validated to be mycoplasma-free, using an ATCC kit. C57BL/6 mice (6-8 week-old female) were obtained from Harlan (Indianapolis, IN), C57BL/6-Tg (TcraTcrb) 1100Mjb/J (OT-1) and CD45.1^+^ mice from the Jackson Laboratories, and CD45.1^+^/OT-1 crossed in our facilities. Female C57BL6/J mice were subcutaneously (s.c.) injected with 1×10^6^ 3LL cells. Tumor volume was determined using calipers and calculated using the formula [(small diameter)^2^ x (large diameter) x 0.5]. All experiments using animals were approved by the GRU-IACUC and performed following GRU animal care facility guidelines.

### Antibodies and reagents

Antibodies against CD3 (clone 145-2C11), CD28 (clone 37.51), CD25 (clone PC61), CD28 (clone CD28.2), CD44 (clone IM7), and CD69 (clone H1.2F3) were obtained from Becton Dickinson Biosciences (BD Biosciences, San Jose, CA). Antibodies against Sca-1 (clone D7) CD62L (clone MEL-14), CCR7 (clone 4B12), CD122 (clone 5H4), and CD127 (clone SB/199) were obtained from eBioscience. Anti-phospho-4E-BP1 (clone 236B4), anti-4E-BP1 (clone 53H11), and anti-TSC2 (D93F12) antibodies were obtained from Cell Signaling Technology (Danvers, MA). Anti-β-actin antibody (clone AC-74) was obtained from Sigma-Aldrich (St. Louis, MO). Nx-hydroxy-nor-Arginine (N-NOHA), L-NG-Monomethylarginine (L-NMMA), and Manganese (III) tetrakis (4-benzoic acid) porphyrin chloride (MnTBAP) were obtained from EMD Millipore (Calbiochem, Gibbstown, NJ). Induction of apoptosis was tested using annexin V Apoptosis Detection Kit (BD Biosciences). Results were expressed as the percentage of annexin V^+^ cells within CD8^+^ cells.

### Isolation of T cells and MDSC

CD8^+^ and CD3^+^ cells were isolated from spleens and lymph nodes of mice using negative isolation kits (Stem Cell Technologies). Cell purity ranged between 95 and 99%, as tested by flow cytometry. MDSC were isolated from tumors previously digested with DNAse and Liberase (Roche USA, Branchburg, NJ) at 37°C for 1 hour, as previously described [25]. Purity ranged from 90 to 99% as measured by flow cytometry. Alternatively, MDSC were generated after culturing bone marrow cells for 4 days with 20 ng/ml G-CSF and GM-CSF [35], followed by sorting of GR-1^+^ cells. iMC were isolated from spleens of mice without tumors, as we described [25]. MDSC were depleted from T cells and MDSC co-cultures by selection of CD11b^+^ cells using magnetic-bead isolation (Stemcell technologies). T cell purity ranged from 96-100% as monitored by flow cytometry.

### Western blot

Cellular extracts isolated from T cells cultured alone or previously co-cultured with MDSC were used for protein detection by immunoblotting. Lysates were electrophoresed in 8% TrisGlycine gels, transferred to PVDF membranes, and immunoblotted against phospho-4E-BP1, 4E-BP1, cyclin D3, cdk4, TSC2, or β-actin. Membrane-bound immune complexes were then detected using ECL western blot detection reagent (GE Healthcare), followed by exposure on X-ray films.

### Measurement of protein translation

T cell protein translation was measured using Click-iT^®^ Labeling kit (Life Technologies). Briefly, T cells were cultured for 48 hours with or without MDSC and in the presence or the absence of specific inhibitors for arginase or iNOS, or a peroxynitrite scavenger [25]. MDSC were then removed and T cells starved for 1 hour of methionine, after which they were labeled for 4 hours with 50 μM Click-iT^®^ L-azidohomoalanine. Protein lysates were harvested and normalized across samples followed by methanol/chloroform precipitation and solubilization in 1% SDS, 50 mM Tris-HCl, pH 8.0. Proteins were treated with Click-IT^®^ Tetramethylrhodamine analysis detection Kit and electrophoresed in 8% TrisGlycine gels, followed by visualization through a BioRad Versadoc.

### Phospho-Zap70 detection

T cells were activated with Dynabeads mouse T-Activator CD3/CD28 beads (Life Technologies) at a cell:bead ratio of 1:1, followed by addition of MDSC to the culture. Four hours later, T cells were stained with anti-CD3 and anti-phospho Zap70 (Y319) antibodies using BD Phosflow kit.

### T cell proliferation assay

T cell proliferation was measured, as we described [25]. For the TSC2 siRNA experiments, T cell proliferation was monitored using eFluor670 (eBioscience).

### siRNA Transfection of T cells

Anti-CD3/CD28-activated CD3^+^ T cells were transfected using Accell SMART pool siRNA from GE Dharmacon. Briefly, T cells were activated and cultured in a 96 well plate overnight in 100 μL Accell media containing 1% FBS and 2 μM of mock or anti-TSC2 siRNA comprised of the following sequences: GCAAUGACUUUGUUUCUAU, GUGUCAUAUGAGAUUGUUC, CUCCUGUCUUUUGAUGAUA, CUAACAGCAUUAAUAUCUU. The following day, MDSC (1:1/2; T cell: MDSC) were added to the cultures and the wells supplemented with complete media for a final volume of 200 μL. Transfection efficiency was monitored using control siRNA labeled with 5 carboxy-fluorescein (FAM) and was determined to be >90%.

### mTOR activity

mTOR activity was assessed using K-LISA™ mTOR Activity Kit from EMD Millipore, according to the manufacturer's instructions. Briefly, T cell lysates were prepared in a lysis buffer comprised of 50 mM Tris HCl, 100 mM NaCl, 50 mM β-glycerophosphate, 10% glycerol (w/v), 1% Tween 20 (w/v), 1 mM EDTA, 20 nM microcystin-LR, 25 mM NaF, and protease inhibitor cocktail Set III. Values were normalized to the maximum mTOR standard, while T cells treated with rapamycin (100 ng/ml) acted as the control for mTOR activity inhibition.

### Statistical analysis

Statistical analyses were carried in GraphPad Prism. Tests were conducted at 5% significance level. Experimental group differences of endpoints were assessed by ANOVA, whereas mean comparisons were carried out with the Tukey procedure or with the Dunnet procedure for comparisons with controls.

## SUPPLEMENTARY MATERIAL FIGURES


