# Supplementary material for: T cells conditioned with MDSC show an increased anti-tumor activity after adoptive T cell based immunotherapy
Source: Oncotarget. 2016 Mar 19;7(14):17565–78. doi: 10.18632/oncotarget.8197 (PMC4951233; doi:10.18632/oncotarget.8197)
Supplement: Supplementary file 1 [file oncotarget-07-17565-s001.pdf]

T cells conditioned with MDSC show an increased anti-tumor activity after adoptive T cell based immunotherapy

Supplementary Material

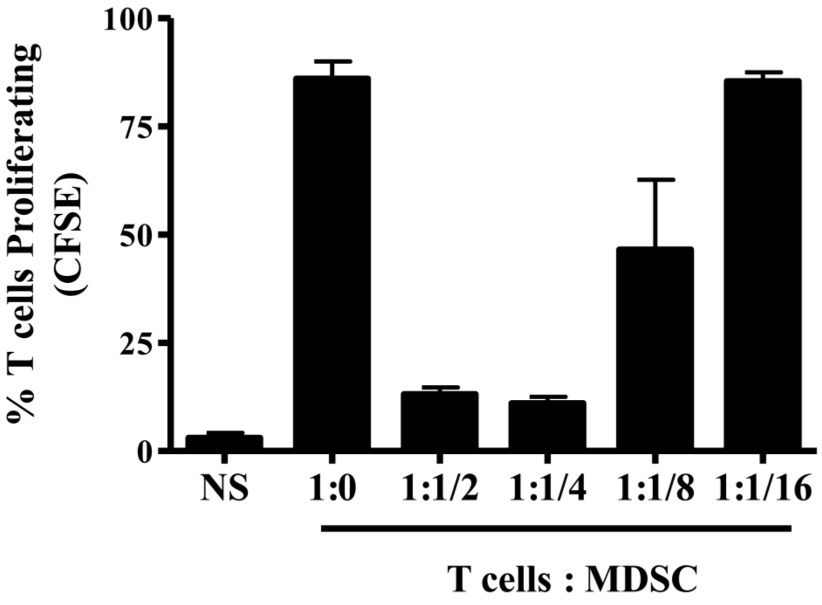

Suppl. Figure 1. MDSC block T cell proliferation.

Anti-CD3/CD28 activated T cells labeled with CFSE were cultured with different numbers of MDSC for 72 hours, after which CFSE dilution was studied by flow cytometry. All data are expressed as mean +/- SEM from 3 experiments. * ** $p < 0.001$

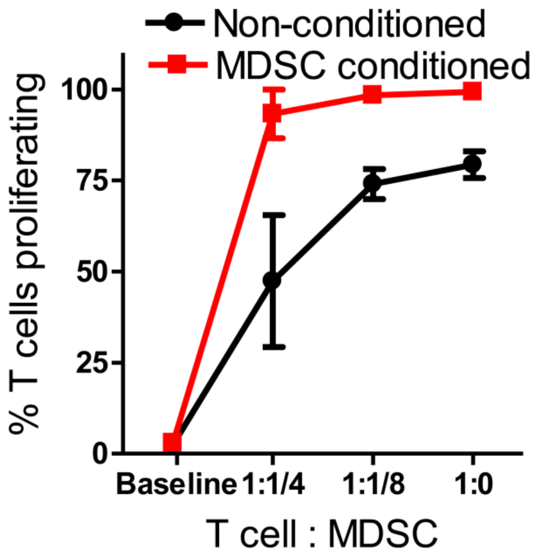

Suppl. Figure 2. MDSC-exposed CD44^{low} T cells become less susceptible to MDSC upon re-exposure.

Activated CD8^{+} T cells were cultured in the presence of MDSC (1:1) for 24 hours, after which the CD8^{+} CD44^{low} and CD8^{+} CD44^{high} T cells were sorted, labeled with CFSE (baseline), and cultured alone or with MDSC (1:1/4; 1:1/8) for additional 72 hours. CFSE dilution was then monitored by flow cytometry. Results are expressed as mean +/- SEM from 3 experiments.

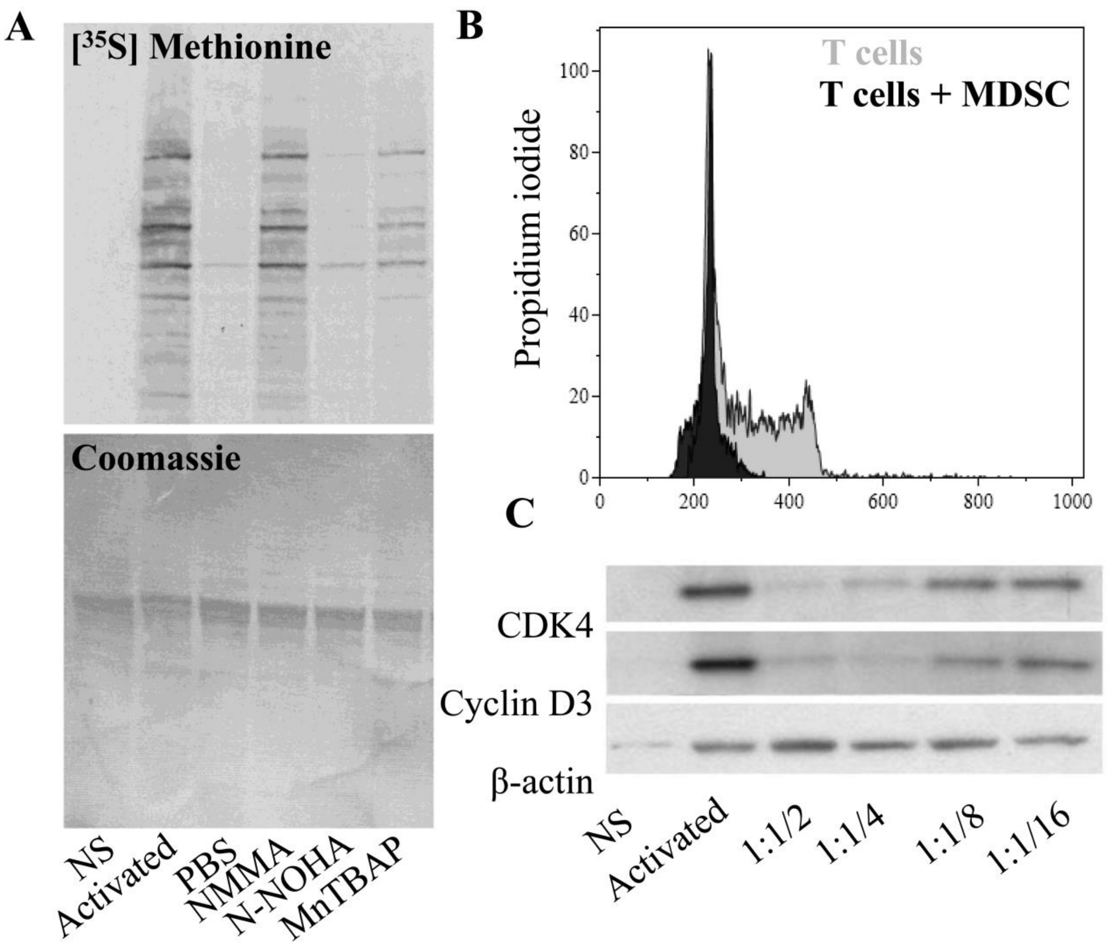

Suppl. Figure 3. MDSC prevent *de novo* protein synthesis in activated T cells

(A) *De novo* protein synthesis was monitored using [^{35}S] Methionine in activated T cells cultured alone (Act) or with MDSC (1:1/2 T cell:MDSC) in the presence of L-NMMA (500µM), N-NOHA (200µM), or MnTBAP (100µM). (B) Representative experiment showing a G_0-G_1 phase arrest in the cell cycle of primed T cells co-cultured with MDSC. Experiments were repeated 3 times. (C) Expression of cyclin D3 and CDK4 proteins was determined by Western blot in activated T cells cultured in the presence of increasing numbers of MDSC.
